# False lumen being larger than true lumen is associated with late aortic events in uncomplicated type B aortic dissection

**DOI:** 10.1093/icvts/ivac003

**Published:** 2022-02-11

**Authors:** Akihito Matsushita, Minoru Tabata, Takashi Hattori, Wahei Mihara, Yasunori Sato

**Affiliations:** 1 Department of Cardiovascular Surgery, Seikeikai Chiba Medical Center, Chiba, Japan; 2 Department of Cardiovascular Surgery, Juntendo University Graduate School of Medicine, Tokyo, Japan; 3 Department of Preventive Medicine and Public Health, School of Medicine, Keio University, Tokyo, Japan

**Keywords:** Uncomplicated Stanford type B Aortic dissection

## Abstract

**OBJECTIVES:**

In uncomplicated type B aortic dissection, a large false lumen (FL) is reportedly a risk factor for late aortic events. However, it is unclear how the relationship between the false and true lumen (TL) diameters affects the dissected aorta. This study aimed to evaluate the impact on clinical outcomes of the FL being larger than the TL.

**METHODS:**

We retrospectively reviewed 111 consecutive patients with uncomplicated acute type B aortic dissection between 2004 and 2018. We divided the patients into group A (FL > TL; *n* = 51) and group B (FL ≤ TL; *n* = 60), and compared the outcomes. The endpoints were aortic events, including surgery for aortic dissection and indication for surgery, and mortality.

**RESULTS:**

The 5-year incidence rates of aortic events were 68.4% in Group A and 33.6% in Group B (*P* = 0.002). The 5-year all-cause mortality rates were 5.3% in Group A and 21.9% in Group B (*P* = 0.003). The multivariable analyses revealed that FL > TL was an independent factor associated with aortic events (adjusted hazard ratio 2.482, 95% confidence interval 1.467–4.198, *P* < 0.001), but had low mortality (adjusted hazard ratio 0.209, 95% confidence interval 0.073–0.597, *P* = 0.003).

**CONCLUSIONS:**

Patients with uncomplicated type B aortic dissection with FL > TL at admission are at increased risk of aortic events but improve mortality compared to patients with FL ≤ TL.

**Clinical trial registration:**

UMIN000036997.

**Figure:**
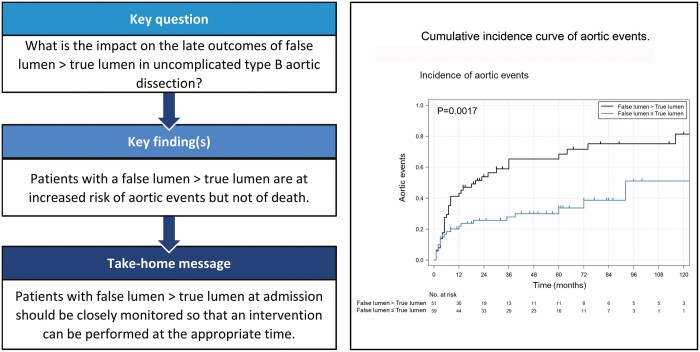

## INTRODUCTION

Poor outcomes for aortic events have been reported for patients with uncomplicated Stanford type B aortic dissection (TBAD) [1]. Almost half of the patients with uncomplicated TBAD who survive the acute phase need aortic surgery for aortic dissection in the chronic phase [2]. The endovascular repair of TBAD—long-term results of the randomized investigation of stent grafts in aortic dissection trial (INSTEAD-XL trial) revealed that the thoracic endovascular aortic repair (TEVAR) group demonstrated a high thrombosis rate of the false lumen (FL) (90.6%) and that the 5-year aorta-related mortality rate was lower in the TEVAR group rather than in the best medical treatment alone group (6.9% vs 19.3%, respectively: *P* = 0.04) [3]. An increasing number of aortic centers now perform pre-emptive TEVAR for high-risk uncomplicated TBAD based on the results of the INSTEAD-XL trial [4]. Many studies have evaluated the risk factors for aortic events in patients with TBAD [4–11]. However, a robust risk model predicting aortic events after uncomplicated TBAD has not been established. In our previous study, we developed a simple risk prediction score system for aortic events in patients with uncomplicated TBAD [5]. It detected high-risk patients as follows: patients with an initial aortic diameter ≥40 mm or patients with an FL diameter larger than true lumen (TL) diameter at admission or patients aged ≥70 years with ulcer-like projections (ULP). The several previous studies from other group also reported about initial aortic diameter [1, 2, 4], aged patients with ULP [6] or FL thickness [7]. However, to the best of our knowledge, no study has investigated the impact of the FL diameter larger than the TL diameter at admission on the late outcomes and aortic dimensions in uncomplicated TBAD.

This study aimed to investigate whether the presence of an FL diameter that is greater than the TL diameter at admission (FL > TL) is associated with aortic expansion and events.

## METHODS

### Ethics statement and study design

The study protocol was in accordance with the Declaration of Helsinki and designed based on the Strobe Statement. And it was approved by the Institutional Review Board of Seikeikai Chiba Medical Center (approval number CMC 2019-5). A waiver of informed consent was obtained.

The study enrolled consecutive patients treated for acute TBAD at 2 centers in Japan between October 2004 and December 2018.

**Figure 1:**
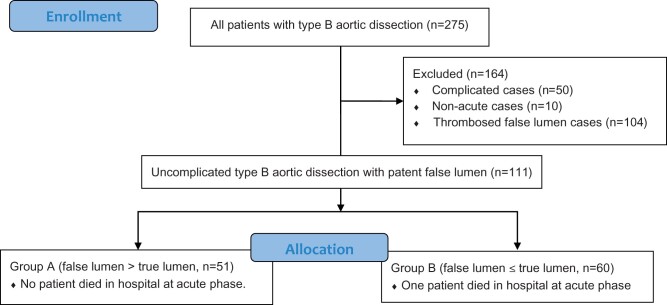
Flow diagram of the entire series of patients with Stanford type B aortic dissection. The study comprised 111 consecutive patients with uncomplicated type B aortic dissection. Patients were divided into 2 groups depending on the false lumen diameter; Group A comprised 51 patients with the false lumen diameter > the true lumen diameter, while Group B comprised 60 patients with the false lumen diameter ≤ the true lumen diameter. CT: computed tomography; CT (+): CT images available.

We excluded patients with non-acute TBAD who were diagnosed 2 weeks or more after the onset of symptoms, patients with complicated TBAD (rupture, impending rupture, malperfusion) [12] and patients with closed thrombosed FL or intramural haematoma. In this study, we retrospectively reviewed consecutive patients with uncomplicated TBAD. The patients were divided into 2 groups based on the initial relationship between the FL and TL diameters on computed tomography (CT) at admission; Group A comprised of patients with FL > TL, while Group B comprised of patients with FL ≤ TL (Fig. 1).

We measured the aortic dimensions before discharge, at 6 months and every year after the onset, and compared the changes in aortic dimensions over time between the 2 groups. The primary endpoints were aortic events after discharge from hospital, including surgery for aortic dissection, indication for surgery [aortic diameter ≥55 mm, rapid aortic enlargement (5 mm per 6 months), saccular aneurysm] and aortic-related mortality; the secondary endpoint was all-cause mortality. We also evaluated the association between aortic events and the FL diameters on CT images obtained at admission and before hospital discharge. Receiver operating characteristic curve analyses were used to assess the discrimination.

The final follow-up data were collected from January 2020 to April 2020 via medical computer records system survey in 2 centers. We also collected the follow-up data for outpatient in another hospital via telephone and mail interviews. CT data were not available for all patients. We checked the follow-up index, defined as the ratio between the investigated follow-up period and the theoretically possible follow-up period up to the pre-specified study and date [13].

### Initial therapy protocol for uncomplicated type B aortic dissection

The diagnosis was made with multiphasic CT, which included non-enhanced, arterial-phase and delayed images. We introduced electrocardiogram-gated CT angiography to accurately identify that the intimal tear or ULP in 2016. Our CT protocol was reported previously [14]. Aortic dissection was classified as Stanford type B if the dissection did not involve the ascending aorta. The present study excluded patients with type B intramural haematoma or thrombosed FL, as it was difficult to distinguish between intramural haematoma and aortic dissection with a totally thrombosed FL. Our initial therapy protocol has been described previously [5]. All uncomplicated TBAD cases were intended to be managed medically, regardless of the FL status or the aortic diameter. We routinely followed up the dissected aorta with contrast-enhanced CT at 1 day and 1–2 weeks after admission, unless the patient had renal dysfunction. Emergency surgery was performed when rapid aortic enlargement or organ malperfusion was identified at any stage; these cases were defined as complicated TBAD. Systolic blood pressure was controlled between 100 and 120 mmHg. After discharge from hospital, the patients continued to receive oral antihypertensive therapy.

### Data collection, definitions and computed tomography measurements

In-hospital data were collected from the medical records. Uncomplicated TBAD was defined according to previous ESC guidelines [12] as follows: no rupture of the dissected aorta; no impending rupture of the dissected aorta (i.e. no continuous symptoms despite optimal medical treatment with analgesic or anti-impulse medication); no malperfusion (i.e. no newly developed symptoms with the presence of FL expansion that impaired the TL flow on CT images). In-hospital mortality was defined as any death before hospital discharge. And aortic-related mortality was defined as all deaths related to the treated pathology, including aneurysm rupture, retrograde progression of the aortic dissection, complications of the operative procedure or clinically suspected of aortic death without other leading cause.

**Figure 2:**
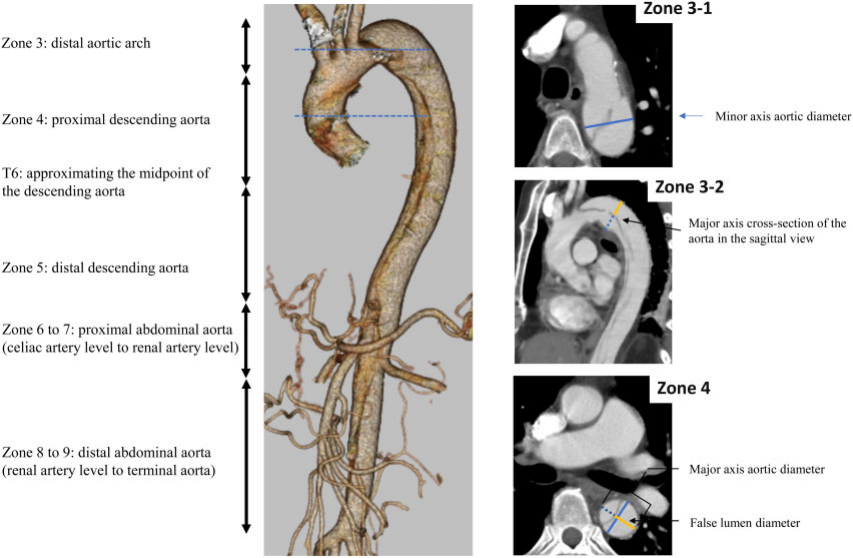
Computed tomography (CT) measurement techniques. Aortic dissection was diagnosed via contrast-enhanced CT in all patients. On CT images obtained at admission, we measured the aortic diameter and checked for false lumen patency on all CT images. The largest major-axis and minor-axis diameters and false lumen diameters were measured at 5 sites based on a previously reported “zone” system, comprising Zone 3 (distal aortic arch), Zone 4 (proximal descending aorta; T6 approximates the midpoint of the descending aorta), Zone 5 (distal descending aorta), Zone 6 to 7 (proximal abdominal aorta; coeliac artery to renal artery) and Zone 8 to 9 (distal abdominal aorta; infrarenal artery). We use a straight line bisecting the centre of the intimal flap and perpendicular to the blood flow angle [15]. We usually used the axial view for the minor-axis diameter measurements. We also used the sagittal view or coronal view for the false lumen diameter and major-axis diameter, as evaluation of the major-axis cross-section of the aorta depends on the patient’s blood flow. The initial aortic diameter was defined as the largest short-axis diameter at any of the 5 sites.

According to Society for Vascular Surgery and Society of Thoracic Surgeons’ suggestion, 3 authors (A.M., T.H. and W.M.) measured the aortic diameter and FL diameter and patency on all CT images [15]. Diameters were measured from the outer aortic wall to the outer aortic wall. We also use a straight line bisecting the centre of the intimal flap and perpendicular to the plane of blood flow as the long-axis diameters. The combined TL and FL diameters will add up to the long-axis aortic diameter. The largest long-axis and short-axis diameters, which are orthogonal to each other and FL diameter, were measured at 5 sites in accordance with the zone system described previously (Fig. 2) [5]. FL > TL (FL diameter/TL diameter + FL diameter > 0.5) was defined from any of the 5 sites on CT at admission. The patients were divided into 2 groups depending on FL diameter as mentioned above. We calculated the expansion rate based on the largest dissected aortic diameter on initial CT versus that on follow-up CT. Patients who underwent surgery for aortic dissection were excluded from the analysis of the expansion rate after surgery.

Patent FL was defined as any contrast effect in the FL at the early or late vascular phase, except for ULP. ULP was defined as focal, well-defined pouches of contrast medium measuring ≤10 mm in length and projecting into the non-communicating FL along the long axis of the aorta. And we regard enlargement of ULP or aneurysmal formation of ULP as a kind of saccular aneurysm.

### Statistical analysis

All our statistical and data reporting have been run according to the *European Journal of Cardio-Thoracic Surgery* guidelines and the *Interactive CardioVascular and Thoracic Surgery* guidelines [16]. For the patient characteristics (Table 1) and CT data (Table 2), categorical data were presented as frequencies and proportions, while continuous variables were presented as the mean and standard deviation or the median and interquartile range (IQR), as appropriate. Univariable analyses were carried out to test the differences of characteristics between Group A and Group B using the *t*-test or the Mann–Whitney *U*-test for continuous variables and Fisher’s exact test for categorical variables. We also tested differences in changing over time for repetitive CT measurements data within individuals using the paired *t*-test. And multivariable analyses were carried out to assess whether baseline FL > TL was associated with aortic events and mortality. All variables reported as major factors associated with outcomes in the literature were entered into the maximum model. Then, only those covariates which did not generate a significant (<10%) change on the hazard ratios (HRs) between FL > TL and outcomes were excluded. Those covariates that significantly changed the HR remained in the final model.

**Table 1:** Characteristics of the patients

Variables	All patients	Group A (FL> TL)	Group B (FL≤TL)	*P*-value
	(*n* = 111)	(*n* = 51)	(*n* = 60)	
Age (years; mean ± SD)	63.9 ± 13.3	56.8 ± 12.6	69.9 ± 10.7	<0.001
Male	81 (73.0%)	39 (76.5%)	42 (70.0%)	0.444
Body mass index	24.5 ± 4.3	25.3 ± 3.8	23.8 ± 4.6	0.058
Hypertension	108 (97.3%)	50 (98.0%)	58 (96.7%)	1.000
Hyperlipidaemia	23 (20.7%)	11 (21.6%)	12 (20.0%)	0.839
Diabetes mellitus	11 (9.9%)	5 (9.8%)	6 (10.0%)	1.000
Haemodialysis	2 (1.8%)	0 (0%)	2 (3.3%)	0.499
Peripheral artery disease	8 (7.2%)	3 (5.9%)	5 (8.3%)	0.724
Coronary artery disease	9 (8.1%)	1 (2.0%)	8 (13.3%)	0.037
Cerebral infarction	8 (7.2%)	1 (2.0%)	7 (11.7%)	0.068
Chronic obstructive pulmonary disease	9 (8.1%)	2 (3.9%)	7 (11.7%)	0.175
De Bakey classification type IIIa	23 (20.7%)	2 (3.9%)	21 (35.0%)	<0.001
De Bakey classification type IIIb	88 (79.3%)	49 (96.1%)	39 (65.0%)	

Data are presented as *n* (%) or mean ± standard deviation.

DeBakeyIIIa: aortic dissection stops above the diaphragm; DeBakeyIIIb: aortic dissection extends below the diaphragm; SD: standard deviation.

**Table 2:** Computed tomography data at onset

Variables	All patients	Group A (FL> TL)	Group B (FL≤TL)	*P*-value
	(*n* = 111)	(*n* = 51)	(*n* = 60)	
Aortic minor-axis diameter at largest site (mm)	37.6 ± 7.4	37.0 ± 7.7	38.1 ± 7.1	0.510
Aortic major-axis diameter at largest site (mm)	39.5 ± 7.4	38.8 ± 7.9	40.1 ± 7.0	0.415
False lumen diameter at largest site (mm)	14.6 ± 6.6	19.7 ± 5.5	10.2 ± 3.7	<0.001
Aortic minor-axis diameter ≥40 mm	38 (34.2%)	16 (31.4%)	22 (36.7%)	0.558
The mean site number of false lumen diameter > true lumen diameter	1.96 ± 1.83	3.21 ± 1.21	0	<0.001
Ulcer-like projection (1 week after onset)	19 (17.1%)	2 (3.9%)	17 (28.3%)	<0.001

Data are presented as *n* (%) or mean ± standard deviation.

For time-to-event outcomes, the cumulative incidence of aortic events was estimated by a competing risk analysis, because death is a competing risk for loss to follow-up, i.e. patients who die can no longer become lost to follow-up. Also, to identify baseline and clinical variables associated with aortic events, competing risk analysis was performed with the Fine–Gray generalization of the proportional hazards model accounting for death as a competing risk with the following covariates: age, male sex, hypertension, chronic obstructive pulmonary disease, De Bakey classification typeIIIb, ULP, initial aortic diameter of ≥40 mm and FL > TL (Table 3). And the mortality was compared using the log-rank test, while the Kaplan–Meier method was used to estimate the absolute risk of each event, and HR and 95% confidence intervals (CIs) were estimated by the Cox proportional hazards model. Also, to identify baseline and clinical variables associated with mortality, multivariable analyses were performed using the Cox proportional hazard model for mortality with the following covariates: age, male sex, body mass index, peripheral artery disease, coronary artery disease, diabetes mellitus, cerebral infarction, De Bakey classification typeIIIb, initial aortic diameter of ≥40 mm and FL > TL (Table 4).

**Table 3:** Multivariable analysis for factors associated with aortic events in acute type B aortic dissection

Variables	Maximum model		Final model	
	Hazard ratio (95% CI)	*P*-value	Adjusted hazard ratio (95% CI)	*P*-value
Age	0.987 (0.969–1.006)	0.171		
Male sex	1.145 (0.612–2.143)	0.671		
Hypertension	1.190 (0.120–11.76)	0.882		
Chronic obstructive pulmonary disease	0.432 (0.097–1.926)	0.432		
De Bakey classification type IIIb	1.292 (0.661–2.526)	0.454		
Ulcer-like projection	0.690 (0.316–1.506)	0.351		
False lumen > true lumen	2.337 (1.370–3.986)	0.002	2.482 (1.467–4.198)	<0.001
Initial aortic diameter of ≥40 mm	3.082 (1.856–5.116)	<0.001	3.236 (1.962–5.338)	<0.001

CI: confidence interval.

**Table 4:** Multivariable analysis for factors associated with all mortality in acute type B aortic dissection

Variables	Maximum model		Final model	
	Hazard ratio (95% CI)	*P*-value	Adjusted hazard ratio (95% CI)	*P*-value
Age	1.033 (0.970–1.100)	0.316		
Male sex	5.997 (0.754–47.679)	0.090	1.442 (0.407–5.106)	0.570
Body mass index	0.911 (0.761–1.090)	0.308		
Peripheral artery disease	1.454 (0.305–6.945)	0.639		
Coronary artery disease	1.368 (0.300–6.235)	0.686		
Diabetes mellitus	1.544 (0.365–6.523)	0.555		
Cerebral infarction	0.208 (0.017–2.590)	0.222		
De Bakey classification type IIIb	1.377 (0.361–5.258)	0.640		
False lumen > true lumen	0.208 (0.052–0.827)	0.026	0.209 (0.073–0.597)	0.003
Initial aortic diameter of ≥40 mm	3.659 (1.116–11.998)	0.032	4.293 (1.658–11.119)	0.003

CI: confidence interval.

All comparisons were planned, and the tests were 2-sided. A *P*-value of <0.05 was considered statistically significant. All statistical analyses were performed using the SAS software program version 9.4 (SAS Institute Inc., Cary, NC, USA), SPSS version 22.0 (IBM Corp., Armonk, NY, USA) and R version 3.00 (R Foundation for Statistical Computing, Vienna, Austria).

## RESULTS

### Patient population

The present study enrolled 275 consecutive patients with TBAD. We excluded 10 patients with non-acute TBAD, 50 patients with complicated TBAD and 104 patients with closed thrombosed FL or intramural haematoma. Almost of these patients with thrombosed FL or intramural haematoma had FL ≤ TL (103/104, 99.0%). We retrospectively reviewed 111 consecutive patients with uncomplicated TBAD and divided the patients into 2 groups; Group A of 51 patients with FL > TL, while Group B of 60 patients with FL ≤ TL (Fig. 1).

### Patient characteristics and outcomes

The patient characteristics are summarized in Table 1. Compared with Group B, Group A was younger (*P* < 0.001), more likely to have De Bakey type IIIb dissection (*P* < 0.001) and less likely to have coronary artery disease (*P* = 0.037).

The CT measurement data at admission are summarized in Table 2. Compared with Group A, Group B were more likely to have ULP (*P* < 0.001). There were no significant differences about duration of stay in the intensive care unit [median 3 days (IQR 2–3.5 days) vs median 2 days (IQR 1–3 days), *P* = 0.226], and duration of hospitalization [median 18 days (IQR 15–22 days) vs median 18 days (IQR 14–21 days), *P* = 0.251]. The in-hospital mortality rate was 1.7% (*n* = 1), as 1 patient in Group B died from respiratory failure associated with chronic obstructive pulmonary disease.

### Aortic diameter and expansion rate

**Figure 3:**
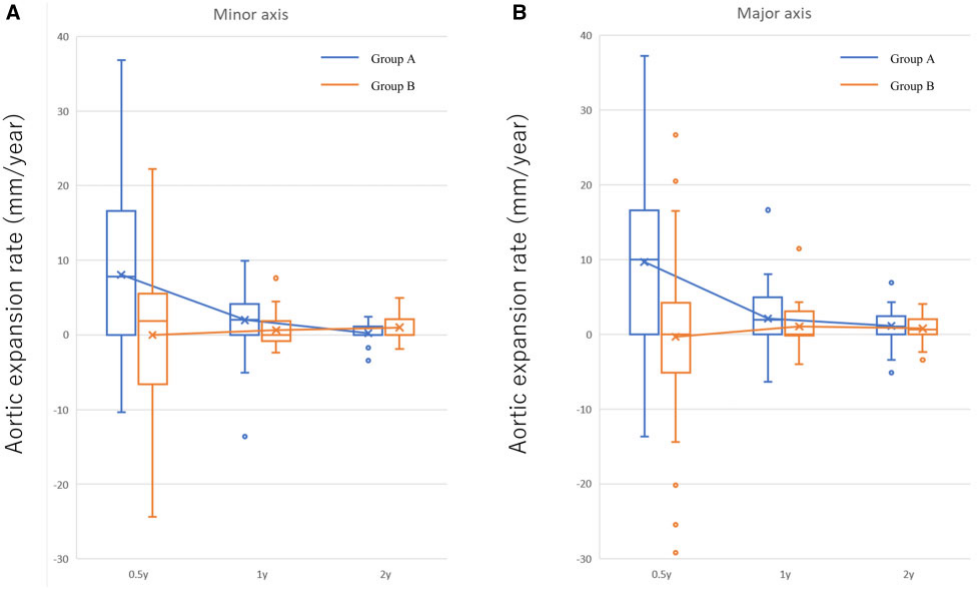
Aortic expansion rate in both groups. (**A**) The minor-axis aortic expansion rate in Group A tended to be faster in the early phase than in the late phase [7.8 (0–16.6) mm in the first 6 months after onset vs 2.0 (0–4.1) mm in the next 6 months, *P* = 0.054]. The aortic growth rate in Group B was almost the same in all phases [1.8 (−6.6 to 5.6) mm in the first 6 months after onset vs 0 (−0.9 to 1.9) mm in the next 6 months, *P* = 0.586]. (**B**) The major-axis aortic expansion rate in Group A was significantly faster in the early phase than in the late phase [9.9 (0–16.6) mm in the first 6 months after onset vs 1.9 (0–5.0) mm in the next 6 months, *P* = 0.004]. The aortic growth rate in Group B did not show the same tendency as in Group A [0 (−5.2 to 4.2) mm in the first 6 months after onset vs 0 (−0.2 to 3.1) mm in the next 6 months, *P* = 0.381].

During follow-up, contrast-enhanced CT was performed. CT data were available for 81.7% (85/104) of patients at 6 months after onset, for 70.8% (63/89) of patients at 1 year after onset and for 70.5% (43/61) of patients at 2 years after onset. The CT measurement data are shown in Supplementary Material, Table S1. The change in both the minor-axis and the major-axis aortic diameter at the largest site was significantly greater in Group A than in Group B at 6 months after onset (*P* < 0.001). The aortic expansion rate in Group A was significantly greater in the first 6 months than in the next 6 months (*P* = 0.004) (Fig. 3); however, the aortic expansion rate in Group B did not show the same tendency.

### Long-term outcomes

**Figure 4:**
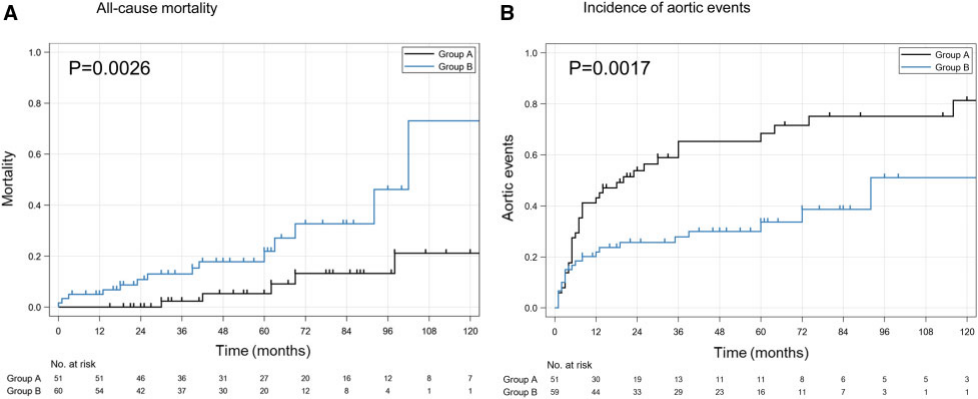
Kaplan–Meier curve and cumulative incidence curve. (**A**) Kaplan–Meier curve of all-cause mortality. (**B**) Cumulative incidence curve of aortic events.

The median follow-up time was 50 months (IQR 27–80 months), and the follow-up rate was 91.9%; 9 patients were lost to follow-up due to a change of address. And the follow-up index was 0.93 (standard deviation: 0.14). The mortality rate was 18.0% (*n* = 20) in all patients. The mortality rate was significantly lower in Group A than in Group B (9.8% vs 25.0%, HR 0.227. 95% CI 0.080–0.644, *P* = 0.005). The aortic-related mortality rate was 7.8% in Group A (due to rupture of a dissected aorta in 2 patients, and newly developed acute type A aortic dissection in 1, cerebral infarction after descending aorta replacement in 1), and 13.3% in Group B (due to rupture of a dissected aorta in 3 patients, newly developed acute type A aortic dissection in 2 patients and sudden unexplained death in 3). The respective proportions of actuarial mortality at 1, 3 and 5 years were 0%, 2.3%, and 5.3% in Group A, and 5.0%, 13.0% and 21.9% in Group B (*P* = 0.003) (Fig. 4A).

Aortic events occurred in 56 (50.5%) patients in the total study cohort during the follow-up period. It was frequently occurred in Group A than Group B [35 patients (68.6%) vs 21 patients (35.0%), HR 2.175, 95% CI 1.260–3.753, *P* = 0.005]. Surgery for aortic dissection was performed in 27 (24.3%) patients. Of these 27 patients, 4 (14.8%) underwent surgery for aortic dissection during the first 6 months after onset, and 13 (48.1%) underwent surgery during the next 6 months. The reasons for surgery included aortic dilatation ≥55 mm in 13 patients, rapid aortic dilatation (>5 mm per 6 months) in 13, dilatation of ULP in 1. Seventeen patients with a dissected aortic aneurysm underwent aortic replacement depending on the aortic dissection site. The descending aorta was replaced in 11 patients. Total aortic arch replacement with a frozen elephant trunk was performed in 5 patients, and a staged additional procedure was performed in 1. Abdominal aortic replacement was performed in a patient. Ten patients underwent TEVAR for dissection of the descending aorta.

During follow-up, 24 patients refused surgery or were deemed inoperable due to their high-risk status, despite having rapid aortic expansion (11 patients, 45.8%), a dissected aorta ≥55 mm (7 patients, 29.2%) or saccular aneurysm (6 patients, 25.0%). Four patients of them (16.7%) died from rupture of a dissected aorta during oral antihypertensive therapy.

The respective incidence rates of aortic events at 1, 3 and 5 years were 43.1%, 65.3% and 68.4% in Group A, and 21.8%, 27.8% and 33.6% in Group B (*P* < 0.001) (Fig. 4B).

### Multivariable analysis

Multivariable analyses revealed that FL > TL at admission was an independent factor associated with aortic events (adjusted HR 2.482, 95% CI 1.467–4.198, *P* < 0.001) (Table 3), but had low mortality (adjusted HR 0.209, 95% CI 0.073–0.597, *P* = 0.003) (Table 4).

### Receiver operating characteristic analysis

Receiver operating characteristic analyses revealed that the cut-off values of the FL diameter for predicting aortic events were 16 mm on CT at admission [sensitivity 62.5%, specificity 74.6%, area under the curve 0.700, 95% CI 0.602–0.798, *P* < 0.001], and 17 mm on CT before discharge (sensitivity 58.9%, specificity 75.9%, area under the curve 0.674, 95% CI 0.573–0.776, *P* < 0.001).

## DISCUSSION

In the present study, we compared the changes in aortic dimensions over time between patients with uncomplicated TBAD with FL > TL versus FL ≤ TL on CT at admission. Patients with uncomplicated TBAD with FL > TL on CT at admission tended to show rapid aortic growth in the first 6 months after onset. The aortic growth rate was not linear in the present study; it was faster in the first 6 months than in the next 6 months. A previous study also reported that the overall aortic growth rate is not linear, with a more prominent initial phase, and that faster aortic growth rate is associated with an increased intervention rate [8].

FL > TL was also a predictor of aortic events in the present study, which is consistent with the findings of a previous study [8]. However, the patients with uncomplicated TBAD in the present study with FL ≤ TL on CT at admission had a slow aortic growth rate. The aortic growth curve is not the same in all patients with uncomplicated TBAD.

Song *et al.* [7] reported that an initial FL diameter of 22 mm was a cut-off value for predicting late thoracic aortic aneurysm (diameter ≥60 mm), with a sensitivity of 100% and a specificity of 76%. However, our receiver operating characteristic analyses did not reproduce their result. Our analyses revealed that the cut-off values of the FL diameter for predicting aortic events were 16 mm on CT at the time of admission and 17 mm on CT before discharge. We think that any cut-off value of the absolute FL diameter cannot be the best predictor of aortic events, and that the presence of FL > TL is easier to evaluate and more reproducible. Tolenaar *et al.* [9] showed that an elliptic configuration of the TL resulting from higher pressurization of the FL is an important predictor of late aortic events. FL > TL is an index incorporating both the FL diameter and the configuration of the FL and TL.

In the present study, multivariable analysis revealed that patients with FL > TL improve mortality compared to patients with FL ≤ TL, and the actuarial mortality rate of the patients with FL > TL at admission was lower than the patients with FL ≤ TL. There were significant differences in patient characteristics between Group A and Group B in this study. Especially, in patients with FL ≤ TL, the average age was 13 years higher than in patients with FL > TL. This classification of the relationship between the FL and TL diameters may affect the discrepancy of the outcomes. In addition, we did not perform surgical treatment for all patients with the aortic event as mentioned above. And we also could not diagnosis operative indications for all patients because some patients were followed without CT data. This diagnosis bias or treatment way also affected the outcomes. Previous studies have reported that the FL diameter is associated with aortic events but not with mortality [1, 10]. Patients with high-risk anatomical forms for aortic events are not always at risk of mortality. Long-term prognosis may be expected in the patients with aortic event underwent appropriate treatment. In our study, the multivariable analysis revealed that initial aortic diameter >40 mm was also an independent factor associated with mortality. We found an aortic rupture in 5 patients with initial aortic diameter >40 mm. Four of them refused surgery or were deemed inoperable due to their high-risk status, despite having a dissected aorta ≥55 mm (3 patients), or saccular aneurysm (a patient). Several previous studies have also showed that the initial aortic diameter is associated with adverse events and mortality [1, 10, 11]. Thus, our results are consistent with these previous studies.

### Limitations

The present study has several limitations. Firstly, this study was a retrospective observational study performed at 2 centers in Japan, and so the sample size and variables were limited. The different CT type was available over the long-period enrolment that changed the possibility to be correct with measurements. And there was a possibility of observer bias without intra, interobserver evaluation. We did not diagnose Marfan syndrome and other genetic diseases, so we could not evaluate whether the outcomes were affected by genetic diseases. Secondly, we excluded TBAD with both a thrombosed FL and type B intramural haematoma, as these conditions were difficult to distinguish. Therefore, we could not evaluate TBAD with completely thrombosed FL. Finally, the follow-up CT data were not available for all patients. Furthermore, we could not follow the natural aortic diameter growth rate in 17 patients (15.3%) because surgery was performed within 1 year after onset. This was not selective drop-out, and the sample size was kept at the previous study level. Moreover, the follow-up index of the present study was over 0.90, which was seemed to be the smaller the risk of selection bias [13]. A large prospective study and external validation studies with high follow-up rates are needed to clarify the factors associated with aortic events or mortality in uncomplicated TBAD.

## CONCLUSIONS

Uncomplicated TBAD patients with FL > TL have different clinical characteristics compared with patients with FL ≤ TL. And the presence of FL > TL on CT at admission is associated with aortic events but had low mortality. Furthermore, these patients with FL > TL at baseline tend to show rapid aortic growth in the first 6 months after onset. Patients with FL > TL on CT at admission should be closely monitored so that intervention can be performed at the appropriate time.

## SUPPLEMENTARY MATERIAL

Supplementary material is available at *ICVTS* online.

**Conflict of interest:** none declared. 

## Data availability statement

The data underlying this article cannot be shared publicly for the privacy of individuals that participated in this study. The data will be shared on reasonable request to the corresponding author.

## Author contributions

**Akihito Matsushita:** Conceptualization; Data curation; Formal analysis; Funding acquisition; Investigation; Methodology; Project administration; Resources; Software; Validation; Visualization; Writing—original draft. **Minoru Tabata:** Conceptualization; Investigation; Methodology; Project administration; Supervision; Validation; Visualization; Writing—original draft. **Takashi Hattori:** Conceptualization; Data curation; Investigation; Methodology; Project administration; Resources; Supervision; Validation; Visualization; Writing—original draft. **Wahei Mihara:** Conceptualization; Data curation; Investigation; Methodology; Project administration; Resources; Supervision; Validation; Visualization; Writing—original draft. **Yasunori Sato:** Conceptualization; Data curation; Formal analysis; Methodology; Software; Supervision; Validation; Visualization; Writing—original draft.

## Reviewer information

Interactive CardioVascular and Thoracic Surgery thanks Luca Bertoglio, Gabriele Piffaretti, Santi Trimarchi and the other anonymous reviewers for their contribution to the peer review process of this article.

## Supplementary Material

ivac003_Supplementary_Material_Table_S1

## Notes

Presented at the 35th Annual Meeting of the European Association for Cardio-Thoracic Surgery, Barcelona, Spain, 13–16 October 2021.

## ABBREVIATIONS

CI: Confidence interval
CT: Computed tomography
FL: False lumen
HR: Hazard ratio
IQR: Interquartile range
TBAD: Type B aortic dissection
TEVAR: Thoracic endovascular aortic repair
TL: True lumen
ULP: Ulcer-like projection
