# Supplementary material for: False lumen being larger than true lumen is associated with late aortic events in uncomplicated type B aortic dissection
Source: Interact Cardiovasc Thorac Surg. 2022 Feb 11;34(6):1132–40. doi: 10.1093/icvts/ivac003 (PMC9714601; doi:10.1093/icvts/ivac003)
Supplement: ivac003_Supplementary_Material_Table_S1 [file ivac003_supplementary_material_table_s1.docx]

Table S1. CT data；Aortic diameter expansion at largest site in early times to 6 months, 6months to 1year, 1 year to 2 year after onset

|  | Major axis diameter | | | Minor axis diameter | | |
| --- | --- | --- | --- | --- | --- | --- |
|  | Group A | Group B | P value | Group A | Group B | P value |
| CT at 6months after onset |  |  |  |  |  |  |
| Diameter of aorta (mm) | 42.7±7.1 | 38.9±7.1 | 0.016 | 40.2±7.2 | 37.0±7.0 | 0.039 |
| Change in the aortic diameter (mm) | 3.0 [0 - 9.0] | 0 [-2.0 – 2.0] | ＜0.001 | 3.0 [0 – 8.0] | 1.0 [-2.3 – 2.3] | ＜0.001 |
| Aortic expansion rate (mm/year) | 9.9 [0 – 16.6] | 0 [-5.2 – 4.2] | ＜0.001 | 7.8 [0 – 16.6] | 1.8 [-6.6 – 5.6] | ＜0.001 |
| CT at 1 year after onset |  |  |  |  |  |  |
| Diameter of aorta (mm) | 42.5±9.1 | 39.3±7.6 | 0.126 | 40.4±8.9 | 37.1±6.9 | 0.111 |
| Change in the aortic diameter (mm) | 1.0 [0 – 2.5] | 0 [-0.3 – 2.0] | 0.179 | 1.0 [0 – 2.5] | 0 [-1.0 – 1.0] | 0.070 |
| Aortic expansion rate (mm/year) | 1.9 [ 0 - 5.0] | 0 [-0.2 – 3.1] | 0.270 | 2.0 [0 – 4.1] | 0 [-0.9 – 1.9] | 0.138 |
| CT at 2 years after onset |  |  |  |  |  |  |
| Diameter of aorta (mm) | 44.5±10.4 | 40.6±7.6 | 0.166 | 41.8±9.2 | 38.9±7.7 | 0.276 |
| Change in the aortic diameter (mm) | 1.0 [0 – 2.0] | 0.5 [0 – 2.0] | 0.518 | 0 [0 – 1.0] | 0 [0 – 1.8] | 0.297 |
| Aortic expansion rate (mm/year) | 1.0 [0 – 2.4] | 0.7 [0 – 2.0] | 0.601 | 0 [0 – 1.1] | 0 [0-2.1] | 0.138 |

Data are presented as mean ± standard deviation or median [interquartile range the 25-th percentile – the 75-th percentile].
